# An analysis of IV tubing in an intensive care unit. is there room for improvement?

**DOI:** 10.1186/2197-425X-3-S1-A555

**Published:** 2015-10-01

**Authors:** F Doesburg, W Dieperink, MWN Nijsten

**Affiliations:** Department of Critical Care, University Medical Center Groningen, Groningen, the Netherlands

## Introduction

ICU patients often receive more than twice the number of intravenous (IV) drugs compared to non-ICU patients. It is up to the ICU nurse to determine how the IV tubing between the infusion pumps and patient should be arranged, preferably using a minimal number of lumens in order to avoid discomfort and catheter-related infections. This is a complex task which is prone to error and involves combining various disposables, such as IV lines and valves, and checking for drug incompatibilities.

## Objectives

The aim of this study was to identify suboptimal arrangements and compatibility errors in the IV tubing at the bedside of adult ICU patients.

## Methods

Between November 4^th^ and November 27^th^ of 2014, the IV tubing arrangements of all patients in our 44-bed ICU were completely mapped out. The resulting schemes included all volumetric and syringe infusion pumps, the administered drugs, flow rates, the type, and placement of catheters, disposables, and their interconnections. A tubing arrangement was considered optimal when a minimal number of lumens was used for a certain combination of drugs without violating compatibility constraints. Optimality of the tubing was determined by checking whether or not all compatible drugs were jointly administered and by identifying unused lumens. An analysis was performed to reveal the joint administration of incompatible drug pairs or combinations with unknown compatibility.

## Results

IV tubing schemes of 160 ICU patients were analyzed. The mean number of infusion pumps was 4.8 ± 2.6 and the overall mean number of lumens was 3.3 ± 1.5. In total, 77 (48%) patients had an already optimal tubing arrangement (mean 2.6 ± 1.5 lumens), and 83 patients (52%) had a suboptimal tubing arrangement (mean 3.9 ± 1.2 lumens). The mean number of lumens of the suboptimal group could significantly be reduced by combining compatible drugs that were administered separately (3.9 vs. 3.4 lumens; p < 0.001). In the suboptimal group a mean of 2.9 ± 2.9 lumens was actually used for the administration of IV drugs and a mean of 1.1 ± 1.0 lumens were maintained as part of a keep-vein-open (KVO) procedure. KVO lumens were observed in 58 (36%) patients, 22 patients (14%) had 2 or more KVO lumens. Notably, 4 (2.5%) patients received an incompatible drug combination and 28 (18%) received a drug combination with an unknown compatibility.

## Conclusions

The IV tubing of ICU patients is often very complex and arranged in a suboptimal fashion, causing more lumens to be used than required, thus increasing infection risk and discomfort. Incompatible and unknown drug combinations were observed, which are both highly undesirable. IV tubing could be optimized by combining compatible drugs more effectively and possibly by looking critically at the number of lumens that need to be kept available for future drug administration.

